# Green Leaf Volatile Profiling Reveals Ripening-Stage- and Tissue-Specific Patterns in Rosaceae Berries

**DOI:** 10.3390/plants15111639

**Published:** 2026-05-27

**Authors:** Dylan Nunnally-Martínez, Amparo Monfort

**Affiliations:** 1 Centre for Research in Agricultural Genomics (CRAG), CSIC-IRTA-UAB-UB, Campus UAB, Edifici CRAG, 08193 Bellaterra, Catalonia, Spain; 2 IRTA, Genomics and Biotechnology, Campus UAB, Edifici CRAG, 08193 Bellaterra, Catalonia, Spain

**Keywords:** strawberry, raspberry, blackberry, aroma, fruit ripening, odor activity values, aldehydes, esters

## Abstract

Green leaf volatiles (GLVs) are key contributors to fruit aroma and plant defense, yet their accumulation during fruit ripening and across tissues remains understudied in *Rosaceae* berries. In this study, GLV profiles of the LOX-HPL pathway were compared between leaves, unripe, half-ripe, and ripe fruit of commercial strawberry (*Fragaria* × *ananassa*), raspberry (*Rubus idaeus*), blackberry (*Rubus* sp.), and woodland strawberry (*Fragaria vesca*) using HS-SPME–GC–MS. Eleven GLVs were identified, exhibiting strong species- and ripening-dependent variation. Odor activity value analysis showed that grassy aldehydes were the main predicted GLV contributors to aroma in ripe raspberry and blackberry samples, as opposed to fruity esters in ripe strawberry samples. However, differences in outdoor growth and harvest conditions should be considered when interpreting interspecies comparisons. Multivariate analyses suggest that GLVs may discriminate between ripening stages and reveal patterns of co-variation between *(Z)*-3-hexenal, *(E,E)*-2,4-hexadienal, and 4-oxohex-2-enal. Berry leaves contained substantially higher total GLVs and distinct compositions compared to fruit, such as the alcohol-rich profile of blackberry leaves, consistent with tissue- and species-associated differences in GLV profiles.

## 1. Introduction

The *Rosaceae* family contains many commercially important berry species, including commercial strawberries (*Fragaria* × *ananassa*), raspberries (*Rubus idaeus*), and blackberries (*Rubus* sp.). Berry fruits are appreciated by consumers for their nutritional value, flavor, and aroma. Aroma is a complex trait involving a blend of tens to hundreds of volatile organic compounds (VOCs). More than 900 VOCs have been identified in the commercial strawberry and the woodland strawberry (*Fragaria vesca*), although only roughly 300 of these volatiles were identified more than once, with an even smaller minority thought to influence consumer preferences [1,2]. The aroma of raspberry and blackberry fruit also involves a complex interplay between hundreds of VOCs from diverse chemical classes, most of which are not odor-active [3,4,5,6]. The distinct classes of VOCs identified in berry fruits include furanones, esters, aldehydes, alcohols, terpenes, sulfur compounds, and ketones, and their accumulation can be influenced by genotype, environment, developmental stage, and postharvest factors [3,7,8,9,10].

Despite the variety of VOCs found in strawberries, previous research has identified a set of 19 key volatile compounds (KVCs) that are the main contributors to the characteristic ripe strawberry aroma [11,12,13]. Five of these KVCs belong to a subset known as green leaf volatiles (GLVs), which are C6 volatiles derived from the LOX-HPL pathway that contribute to green, fresh, and fruity notes. Additionally, the GLVs *(E)*-2-hexenal and hexyl acetate have been positively correlated with sweetness and consumer enjoyment in commercial strawberries, further demonstrating the importance of GLVs for flavor perception [1]. However, the opposite was true for the GLV *(E)*-2-hexenyl acetate, which, alongside the GLVs *(E)*-2-hexen-1-ol and 1-hexanol, was also positively correlated with sourness. In raspberry fruit, research has shown that 18 VOCs mainly contribute to aroma, three of which are GLVs [6]. GLVs also influence aroma in raspberry-derived products other than fresh fruit; of the 14 odor-active VOCs identified in raspberry juice, four are GLVs [14]. While there is comparatively less research on blackberry aroma, the GLV hexanal is a major contributor to aroma in the variety “Marion” and, in the variety “Chickasaw”, the GLVs hexenal and *(E)*-2-hexenal are important volatiles depending on location [15,16].

The investigation of berry aroma has primarily focused on ripe fruit, while there are fewer studies on the dynamic changes in aroma that occur during the ripening process. An improvement in the understanding of how VOCs such as GLVs evolve in berry fruit could lead to the identification of key biomarkers, ensuring an ideal aroma and flavor at harvest and thus providing higher quality berries to consumers [17]. Previous work on the commercial strawberry cultivar Candonga demonstrated that the ester GLV hexyl acetate and the aldehyde GLVs hexanal and 2-hexenal increase during ripening [18]. However, hexanal and 2-hexanal decreased during ripening in three Chinese commercial strawberry cultivars [19]. These contradictory results illustrate the need for further research to determine if GLVs could be useful as ripening biomarkers across several cultivars. In raspberries, changes in GLVs during ripening appear to be cultivar-specific, although *(E)*-2-hexenal was one of few VOCs that generally decreased during ripening [5]. In the blackberry species *Rubus fruticosus*, the alcohol GLV *(Z)*-3-hexen-1-ol was found to be correlated strongly with the unripe stage, while the other quantified GLVs did not show linear patterns during ripening [17]. Likewise, most GLVs identified in the blackberry species *Rubus ulmifolius* did not follow a clear pattern during ripening, except for *(E)*-2-hexen-1-ol, which increased [7].

Besides impacting fruit aroma, GLVs are known to play an important role in ecological interactions and are found in other tissues such as leaves [20,21]. When leaves are mechanically wounded or fed upon by herbivores, many plants emit high quantities of GLVs, including *Arabidopsis thaliana*, maize, and rice [22,23,24,25,26]. Furthermore, GLVs have been shown to play a key role in plant–plant communication, defense priming against herbivores, and interactions with fungal pathogens [27,28,29,30]. In addition, leaf VOCs including the GLV *(E)*-2-hexenal have been used as markers to discriminate between different varieties of the same fruit species [31]. Although leaf GLVs have many important functions, there is less research regarding VOCs and GLVs in leaves of berry plants when compared with research on berry fruits. When leaves of the commercial strawberry cultivar Albion were exposed to high UV-C irradiation, five of the VOCs that showed the most significant increase were GLVs [32]. In leaves of several *Rubus* species, including the blackberry species *R. fruticosus*, GLVs are a key part of the response to herbivory from the raspberry bud moth [33]. Lastly, the GLV 2-hexenal was identified as the largest constituent of raspberry leaf VOCs from a hexanoic extract [34].

While previous studies have characterized VOCs including GLVs in several berry species, the use of several different methodologies makes it difficult to make direct comparisons between species, ripening stages, varieties, and tissues reported in the literature. In this study, we used a standardized SPME-GC-MS method to quantify and compare GLVs in unripe fruit, half-ripe fruit, ripe fruit, and leaves of one woodland strawberry cultivar, three commercial strawberry cultivars, three blackberry breeding lines, and three raspberry breeding lines. This study aimed to further the understanding of aroma in berries as they ripen, identify potential candidate biomarkers related to ripening, compare leaf GLVs with fruit GLVs and between species, and inform future research focused on the biosynthetic regulation of GLVs in berries from the *Rosaceae* family, which is yet to be fully elucidated.

## 2. Results

### 2.1. Evolution of GLV Compounds During Berry Fruit Ripening

Unripe, half-ripe, and ripe berries from one cultivar of woodland strawberry (*Fragaria vesca*), three cultivars of commercial strawberry (*Fragaria* × *ananassa*), three breeding lines of blackberry (*Rubus* sp.), and three breeding lines of raspberry (*Rubus idaeus*) were analyzed for GLV content. The same SPME-GC-MS methodology was applied to all fruit samples, enabling direct comparisons of GLV profiles across species, ripening stages, cultivars, and breeding lines. In total, 11 GLVs were detected in berry fruits of varying ripening stages, including aldehydes, alcohols, and esters; Table 1. The GLVs identified showed unique profiles throughout ripening and between different species, with abundance varying several orders of magnitude in some cases; Supplementary Table S1. Interpretations of comparisons between species should take into account differences in outdoor growth and harvesting conditions. While non-GLV aldehydes, alcohols, esters, terpenes, and ketones were detected in the headspace of both fruit and leaf samples, only C6 GLV aldehydes, alcohols, and esters were quantified in this study.

**Figure 1:**
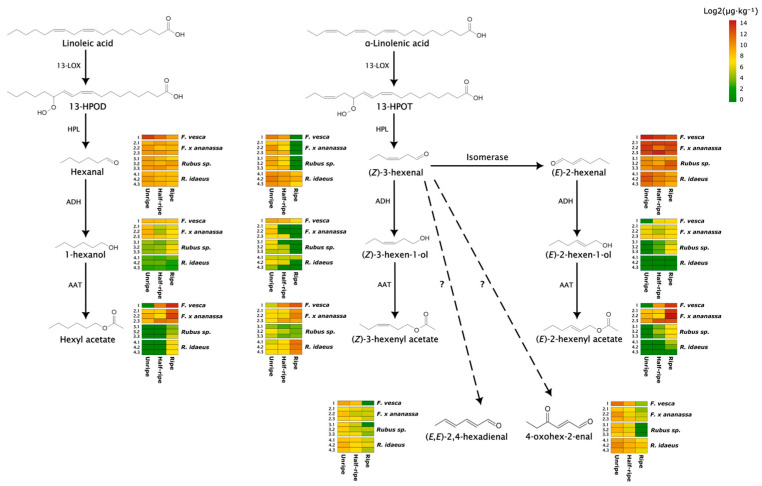
GLVs content in ripening berries overlayed with the LOX-HPL pathway. Mean GLV normalized abundances (μg kg^−1^ relative to 3-hexanone) from unripe, half-ripe, and ripe developmental stages of fruit from various lines of woodland strawberry (*F. vesca*, 1 = Reine de Vallées), commercial strawberry (*F.* × *ananassa*, 2.1 = Dover, 2.2 = Starlette, 2.3 = Tudla), blackberry (*Rubus* sp., 3.1 = RM-167, 3.2 = RM-215, 3.3 = RM-276), and raspberry (*R. idaeus*, 4.1 = RF-055, 4.2 = RF-060, 4.3 = RF-076). Dashed line arrows represent non-canonical GLV whose biosynthesis has not been fully elucidated. Values represent the mean of n = 3 technical replicates of a single pooled sample.

In all unripe and half-ripe berries selected for analysis, ripe blackberries, and ripe samples of the strawberry cultivar Tudla, the most abundant compound was the aldehyde *(E)*-2-hexenal, ranging from a normalized abundance of 584 to 23,892 µg kg^−1^ on average; Supplementary Table S2. The most consistent ripening-dependent pattern of *(E)*-2-hexenal was observed in raspberry samples, where it decreased 80.28% to 82.09% from the unripe stage to the ripe stage depending on the breeding line. In contrast, changes in *(E)*-2-hexenal during ripening were not consistent between breeding lines or cultivars for the selected commercial strawberry and blackberry samples. Selected *Fragaria* samples generally had higher *(E)*-2-hexenal abundance than *Rubus* samples at the same ripening stage, except for the half-ripe commercial strawberry cultivar Starlette; Figure 1. The aldehyde hexanal maintained a relatively stable profile throughout ripening, except for the woodland strawberry cultivar Reine de Vallées, which saw a 92.82% decrease. Unripe Reine de Vallées had the highest normalized abundance of hexanal by far with 12,402 µg kg^−1^. When comparing selected samples from the other three species, hexanal normalized abundances were quite similar between commercial strawberry (410 to 1393 µg kg^−1^), blackberry (242 to 1457 µg kg^−1^), and raspberry (375 to 1088 µg kg^−1^) throughout all three ripening stages. Unlike *(E)*-2-hexenal and hexanal, the aldehyde *(Z)*-3-hexenal and the putatively assigned aldehydes *(E,E)*-2,4-hexadienal and 4-oxohex-2-enal consistently decreased from the unripe to ripe stage across all four species; Figure 1. In general, *(Z)*-3-hexenal, *(E,E)*-2,4-hexadienal, and 4-oxohex-2-enal had similar normalized abundances between all four species at the unripe and half-ripe stages, while larger differences emerged at the ripe stage. For instance, *(Z)*-3-hexenal was only present in ripe raspberries and was not detected in ripe fruit of the other three species sampled, while 4-oxohex-2-enal was not detected in ripe blackberries but was present in the ripe fruit of the other three species sampled.

In comparison with their precursor aldehydes from the different branches of the LOX-HPL pathway, the GLV alcohols had lower normalized abundances. For example, 1-hexanol ranged from a normalized abundance of 13 to 196 µg kg^−1^ in blackberry samples, much lower than the precursor aldehyde hexanal; Supplementary Table S2. As with the aldehydes, ripening- and species-dependent patterns for GLV alcohols vary on a compound-by-compound basis. In the selected woodland and commercial strawberry samples, 1-hexanol normalized abundance did not drastically change between unripe and ripe fruit. This is also the case for selected raspberry samples, although they contain much lower abundance than the strawberry samples. In blackberry samples, 1-hexanol abundance was similar in unripe and half-ripe berries and peaked at the ripe stage. The increase from the unripe stage to the ripe stage was 196% for the RM-167 breeding line, 630% for RM-215, and 890% for RM-276. The GLV alcohol *(Z)*-3-hexen-1-ol decreased during ripening for all berry samples. The woodland strawberry cultivar Reine de Vallées had the highest normalized abundance of *(Z)*-3-hexen-1-ol at all three ripening stages, decreasing from 922 to 140 µg kg^−1^ during ripening. Alongside the raspberry breeding line RF-055, these two were the only berry samples containing *(Z)*-3-hexen-1-ol in the ripe stage. The alcohol *(E)*-2-hexen-1-ol showed the opposite trend, as it increased during ripening in samples of woodland strawberry and blackberry and stayed relatively stable in commercial strawberries. However, no *(E)*-2-hexen-1-ol was detected in raspberry samples, making it the only GLV to not be present in any ripening stage or breeding line for a particular species.

Lastly, the GLV esters showed a general increase during ripening, except for the commercial strawberry cultivar Tudla and *(Z)*-3-hexenyl acetate in the blackberry samples. For hexyl acetate and *(E)*-2-hexenyl acetate, clear differences can be seen between the species sampled; Figure 1. Both esters were abundant in unripe commercial strawberries but absent in unripe woodland strawberries, raspberries, and blackberries. However, in the half-ripe and ripe stages, normalized abundances of these two GLV esters were similar between commercial and woodland strawberry samples. Hexyl acetate was the most abundant compound in ripe fruit from the woodland strawberry cultivar Reine de Vallées (17,601 µg kg^−1^), whereas *(E)*-2-hexenyl acetate was the most abundant GLV in ripe fruit of the commercial strawberry cultivars Dover (13,856 µg kg^−1^) and Starlette (31,451 µg kg^−1^), with Starlette exhibiting the highest normalized abundance of any compound in ripe fruit samples. For all ripe raspberry samples, the ester *(Z)*-3-hexenyl acetate was the predominant GLV, ranging from a normalized abundance of 1892 µg kg^−1^ in the RF-076 breeding line to 2732 µg kg^−1^ for the RF-060 breeding line.

### 2.2. Odor Activity Value Estimates and Predicted Influence of GLVs on Berry Fruit Aroma

The odor activity values of GLV in ripening berry fruits were calculated by dividing the normalized abundance of a compound (μg kg^−1^ relative to 3-hexanone) by its odor threshold, which can be compared in Table 2. Due to the semi-quantitative nature of the analysis, OAVs and between-group comparisons are approximate and exploratory. From eleven GLVs detected in all fruits, only nine were predicted to be odor-active (OAV > 1) in at least one berry sample, with 1-hexanol being the exception; there was no odor threshold for 4-oxohex-2-enal available in the literature. Out of the nine odor-active compounds, only hexanal and *(E)*-2-hexenal were estimated to be odor-active in all fruit samples and ripening stages.

**Figure 2:**
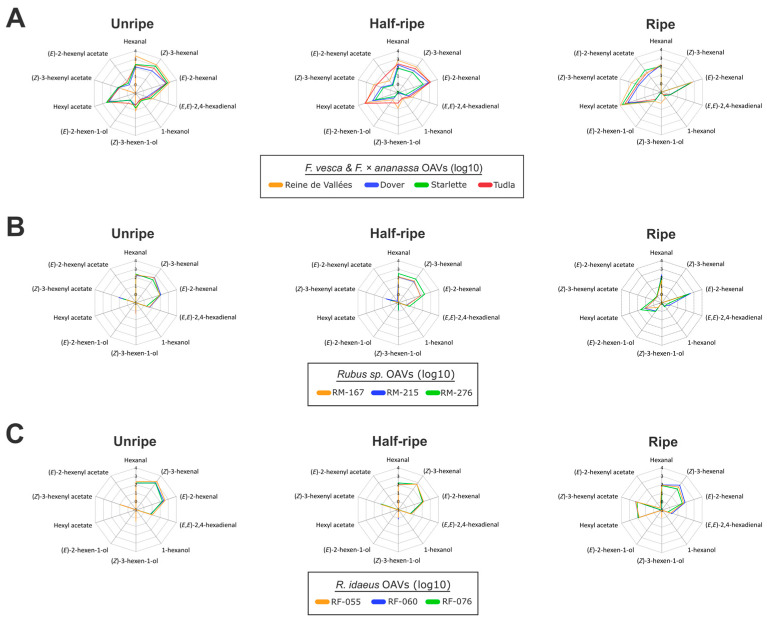
Comparison of OAVs between fruits of the same species in different developmental stages. Radar plots illustrating log10 odor activity values (OAVs) from unripe, half-ripe, and ripe developmental stages of fruit from various lines of (**A**) strawberry (*F. vesca* & *F.* × *ananassa*), (**B**) blackberry (*Rubus* sp.) and (**C**) raspberry (*R. idaeus*).

In unripe berries of all four species, the dominant predicted GLV aroma contribution came from the aldehydes *(E)*-2-hexenal, hexanal, and *(Z)*-3-hexenal; Figure 2. These aldehydes are mainly associated with green, grassy, and fatty notes. In unripe commercial strawberry samples, *(E)*-2-hexenal had the highest OAV of any compound, ranging from 557 in Dover to 811 in Starlette; Table 2. Unripe raspberry and blackberry samples had a similar GLV aroma profile, albeit with blackberries having lower aldehyde OAVs and likely less green and grassy aroma.

In half-ripe fruits, the variation in predicted GLV aroma between cultivars of commercial strawberry became more evident; Figure 2. Tudla, in particular, was quite different to half-ripe Dover and Starlette as it had higher OAVs for all major odor-active compounds. For both Tudla and woodland strawberry cultivar Reine de Vallées, hexyl acetate was the largest contributor to GLV aroma in half-ripe fruit, with OAVs of 1510 and 1066, respectively. In contrast, half-ripe strawberries of Starlette and Dover still had stronger green and grassy GLV aroma profiles defined by aldehydes. The GLV aroma profiles of blackberry and raspberry breeding lines did not change drastically between unripe and half-ripe stages; Figure 2.

The ripe fruit of woodland and commercial strawberry cultivars shared a set of seven predicted odor-active compounds, with hexyl acetate, *(Z)*-3-hexenyl acetate, *(E)*-2-hexenyl acetate, hexanal, and *(E)*-2-hexenal being the largest expected contributors to the GLV aroma; Table 2. For all ripe strawberry fruit, hexyl acetate had the biggest impact on GLV aroma, contributing fruity and sweet notes. Hexyl acetate OAVs spanned between 1088 in Tudla to 8800 in Reine de Vallées, the highest OAV of any compound in any sample. Besides the increase in GLV esters and associated fruity aromas compared with half-ripe and unripe strawberries, there was also a decrease in GLV aldehydes associated with green notes, in particular *(Z)*-3-hexenal. The largest difference in OAVs between the different cultivars of woodland strawberry and commercial strawberry pertained to the three GLV esters, while hexanal and *(E)*-2-hexenal OAVs were quite similar between all four cultivars; Table 2.

In the ripe stage, blackberry fruit had between three and five predicted odor-active GLVs. Relative to the half-ripe and unripe fruit, ripe blackberries began to have fruity aroma contribution from hexyl acetate and completely lost the green aroma contribution from *(Z)*-3-hexenal; Figure 2. However, there was a much lower impact of GLV esters on aroma overall than strawberries, with the aldehydes hexanal or *(E)*-2-hexenal still having the highest OAVs. Ripe raspberry samples had either five or six odor-active GLVs. Unlike the other analyzed berries, ripe raspberry fruit maintained major aroma contribution from *(Z)*-3-hexenal, ranging from 121 for the RF-076 breeding line to 420 for RF-060. Additionally, ripe raspberry samples had higher fruity aroma contribution from the esters *(Z)*-3-hexenyl acetate and hexyl acetate than blackberries.

### 2.3. Total and Proportional GLV Composition in Berry Fruits and Leaves

**Figure 3:**
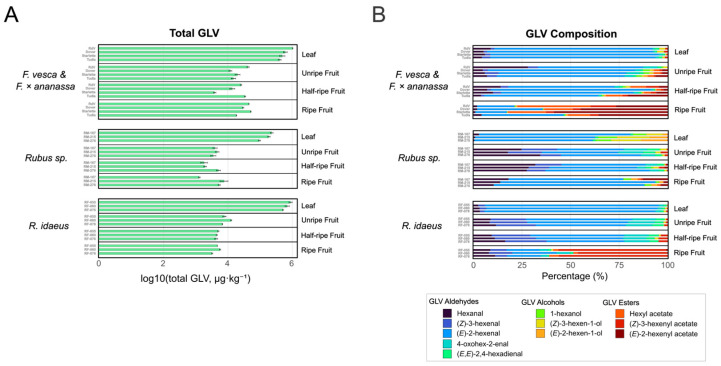
Total and relative GLV composition in unripe fruit, half-ripe fruit, ripe fruit, and leaves of selected lines of woodland strawberry, commercial strawberry, blackberry, and raspberry. (**A**) Bar chart illustrating total GLVs (log10 µg kg^−1^ relative to 3-hexanone). Values represent the mean ± standard deviation of n = 3 technical replicates of a single pooled sample. (**B**) Proportion of each GLV compound relative to the total GLVs.

GLV abundances were quantified in leaves of the same cultivars and breeding lines of woodland strawberry, commercial strawberry, blackberry, and raspberry and compared to GLV abundances in berry fruits. In Figure 3A, the log10 total GLVs are shown for leaves, unripe, half-ripe, and ripe fruit. It is quite evident that the leaves had substantially more total GLVs than the fruit of the same cultivar/breeding line, Supplementary Table S2, although matrix-dependent extraction efficiency cannot be fully excluded between fruit and leaf samples.

The leaves of the woodland strawberry cultivar Reine de Vallées had the highest total GLVs overall with an average relative abundance of 1,080,455 µg kg^−1^, approximately 23× more than ripe fruit of the same cultivar; Supplementary Table S2. Following this, raspberry leaves had the second-highest total leaf GLVs, with relative abundance ranging from 541,335 to 933,133 µg kg^−1^ in RF-076 and RF-055, respectively. Commercial strawberry leaves had a slightly lower total GLV relative abundance range from 428,254 µg kg^−1^ in Tudla to 639,603 µg kg^−1^ in Dover. Blackberry had the lowest total leaf GLV relative abundances by far, spanning from 99,848 µg kg^−1^ in RM-276 to 240,658 µg kg^−1^ in RM-167.

The changes in total GLVs in different fruit ripening stages were quite varied. In woodland strawberry and commercial strawberry, there was not a clear pattern of total GLV changes during ripening. Starlette ripe fruit had the highest average total relative abundance of GLVs of any strawberry sample with 53,871 µg kg^−1^. Likewise, there was not a clear pattern of total GLV evolution during ripening in blackberry fruit. For the raspberry fruit, total GLVs peaked at the unripe stage for all three breeding lines. In general, woodland and commercial strawberry fruit had more total GLV than blackberry or raspberry fruit of the same ripening stage; Supplementary Table S2.

Furthermore, the proportional GLV composition of sampled leaves was different than their fruit counterparts; Figure 3B. For instance, leaves did not contain the GLV esters *(E)*-2-hexenyl acetate and hexyl acetate. Aldehyde GLVs, especially *(E)*-2-hexenal, clearly dominate the GLV profiles of raspberry (97–99%) and strawberry (94–99%) leaves. While *(E)*-2-hexenal was still the majority GLV compound in blackberry leaves, they had a unique GLV profile, with alcohols making up 17 to 35% of total GLV. Blackberry leaves also had a much smaller proportion of *(Z)*-3-hexenyl acetate and putatively assigned 4-oxohex-2-enal when compared with the other sampled leaves.

The evolution of GLV composition during berry fruit ripening can be seen in Figure 3B. In woodland and commercial strawberry fruit, aldehydes make up a majority of the unripe (83–96%) and half-ripe (66–93%) GLV composition, while esters were the majority in ripe fruit (51–82%); Supplementary Table S3. In the fruit of the blackberry samples, aldehydes make up the majority of GLV composition in the unripe (93–96%), half-ripe (91–97%), and ripe (77–93%) stages, while GLV esters peaked in the ripe stage at 2.5–15%. When compared with strawberry fruit, hexanal, *(Z)*-3-hexenal, and putatively assigned *(E,E)*-2,4-hexadienal and 4-oxohex-2-enal, made up a larger portion of the total aldehydes in blackberry and raspberry fruit samples. In raspberry fruit, aldehyde GLVs dropped from 97–98% of total GLV in the unripe stage to 39–51% in the ripe stage. The percentage of esters increased during ripening and peaked at the ripe stage at 49–61%. For all species and ripening stages, GLV alcohols were minor components, never exceeding 7% of total GLVs. Blackberry fruit were the only samples where GLV alcohols peaked in the ripe stage.

In commercial strawberry fruit, *(E)*-2 GLV isomers made up the majority of GLVs in all samples (68–85%) and were proportionally quite stable during ripening; Supplementary Table S3. The proportion of *(Z)*-3 isomers decreased from 5–10% in unripe fruit to 4% in ripe fruit. The fruit of woodland strawberry cultivar Reine de Vallées had a lower proportion of *(E)*-2 isomers (46–60%), while *(Z)*-3 isomers increased from 7 to 13%. As ripening progressed in blackberry fruit, the proportion of *(Z)*-3 isomers decreased from 13–22% in the unripe stage to 0.5–1% in the ripe stage, while *(E)*-2 isomers increased from 34–41% to 75–83%. In raspberry fruit, *(Z)*-3 isomers made up a larger proportion of the GLV composition when compared with the other species. *(Z)*-3 isomers increased during raspberry ripening, starting at 20–23% and peaking at 58–64% in the ripe stage. Conversely, the proportion of *(E)*-2 isomers decreased during ripening from 46–54% in the unripe stage to 17–23% in the ripe stage.

### 2.4. Multivariate Analysis of GLV in Berry Fruits and Leaves

**Figure 4:**
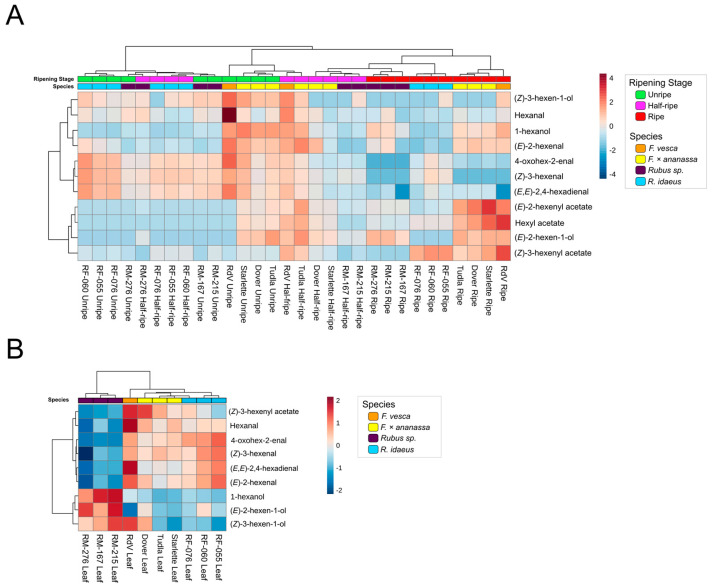
Hierarchical clustering heatmaps. (**A**) Fruit GLVs during different ripening stages and (**B**) leaf GLVs across various berry species and lines.

In order to identify broader patterns in GLV composition and examine how they shape the relationship between sample groups and compounds, hierarchical clustering analyses were conducted. However, as each data point represents a single pooled sample, the patterns reflect differences between composite samples and should be interpreted as exploratory rather than statistically validated groupings. The first hierarchical clustering analysis revealed three main clusters of GLV compounds in fruit samples; Figure 4A. The first consisted of *(Z)*-3-hexen-1-ol, 1-hexanol, hexanal, and *(E)*-2-hexenal, the second consisted of the aldehydes *(Z)*-3-hexenal, 4-oxohex-2-enal, and *(E,E)*-2,4-hexadienal, and the third contained all three GLV esters and *(E)*-2-hexen-1-ol. The strongest correlation within a cluster of compounds was between *(Z)*-3-hexenal, 4-oxohex-2-enal, and *(E,E)*-2,4-hexadienal; Supplementary Table S4.

Regarding clustering of fruit samples, the analysis revealed several main clusters spanning ripening stages and species; Figure 4A. Unripe blackberries and raspberries, half-ripe raspberries, and one half-ripe blackberry sample (RM-276) formed a clear cluster, which was characterized by high content of *(Z)*-3-hexenal and the putatively assigned compounds 4-oxohex-2-enal and *(E,E)*-2,4-hexadienal. The next cluster of unripe strawberries and some half-ripe strawberries contained a diverse amount of GLVs, with the woodland strawberry cultivar Reine de Vallées forming an outgroup due to its very high hexanal content. The third main cluster contained ripe raspberries and blackberries, the half-ripe blackberry samples RM-167 and RM-215, and half-ripe commercial strawberry samples Dover and Starlette; the ripe samples in the third cluster form subcluster groups. The ripe blackberry subcluster was defined by high *(E)*-2-hexen-1-ol and 1-hexanol, as well as low *(Z)*-3-hexenal, 4-oxohex-2-enal, and *(E,E)*-2,4-hexadienal. The ripe raspberry subcluster was characterized by higher levels of *(Z)*-3-hexenal and 4-oxohex-2-enal relative to other ripe berries and by *(Z)*-3-hexenyl acetate relative to other GLV esters. The fourth main cluster consisted of ripe woodland and commercial strawberry samples, which were defined by high amounts of all three GLV esters, 1-hexanol, and *(E)*-2-hexenal, as well as low levels of *(Z)*-3-hexenal and putatively assigned 4-oxohex-2-enal and *(E,E)*-2,4-hexadienal.

For the leaf samples, the GLV compounds formed two main clusters, the first of which contained all three GLV alcohols and the second consisting of the remaining GLVs; Figure 4B. There are two main sample clusters, with the first consisting of blackberry leaves, which are characterized by their high alcohol GLVs and relatively lower amounts of other GLVs. The second cluster contained the leaves of raspberry, woodland strawberry, and commercial strawberry samples, which are characterized by their low GLV alcohols, with the rest of the GLVs being higher relative to the blackberry cluster.

**Figure 5:**
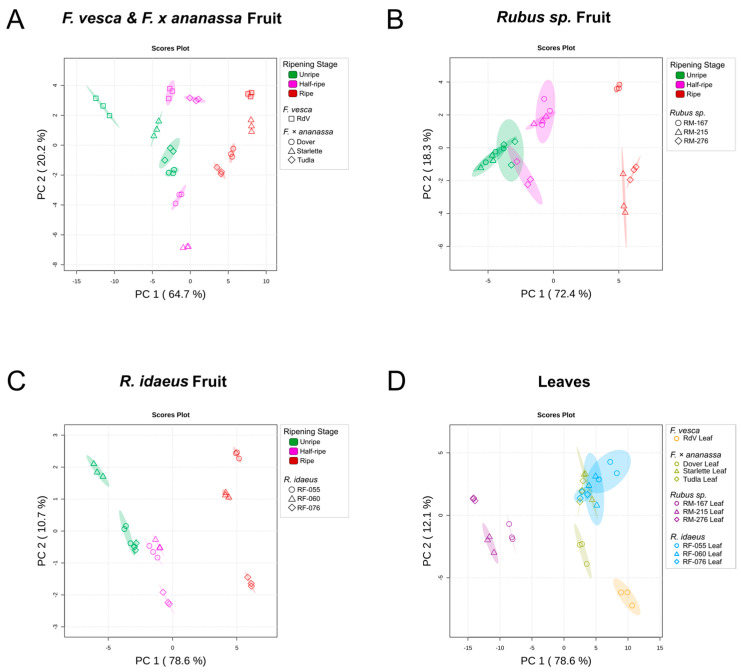
PCA plots of fruit by species and leaves. Panels illustrate all ripening stages of selected samples: (**A**) woodland and commercial strawberry, (**B**) blackberry and (**C**) raspberry fruit; panel (**D**) illustrates leaf GLVs in the aforementioned samples, with each color representing a distinct species.

Principal component analysis of GLV profiles within each berry species at different ripening stages revealed patterns of metabolic differentiation; Figure 5A–C. For all analyzed species, the first component of the PCA represented a majority of the variance, ranging from 64.7% for the strawberry fruit to 78.6% for the raspberry fruit. In the raspberry fruit PCA, the unripe, half-ripe, and ripe samples all formed distinct clusters. In the strawberry and blackberry fruit PCAs, the ripe samples formed a distinct cluster while there was some overlap between unripe and half-ripe samples for blackberry fruit. Principal component analyses of different species at the same ripening stage were also performed, with the first component explaining 49.6% of the variance in the unripe stage, 69.5% in the half-ripe stage, and 60% in the ripe stage; Supplementary Figure S1. While there was some overlap in the unripe and half-ripe PCAs, strawberries, raspberries, and blackberries formed clearly distinct clusters in the ripe stage. For the PCA of leaf samples, the first component also represented a majority of variance (78.6%), with blackberries forming a clear cluster in comparison with the overlap of raspberry and commercial strawberry leaves stages; Figure 5D.

## 3. Discussion

Green leaf volatiles derived from the LOX-HPL pathway are known to contribute to the perception of green, fruity, and fresh notes in fruit aroma and also function as important ecological signals in both fruit and leaves. Despite their relevance, the dynamics of GLVs in *Rosaceae* berries remain relatively underexplored, and direct cross-species comparisons provide an opportunity to improve the understanding of their regulation. The differences in GLV abundances between strawberry, blackberry, and raspberry fruit samples discerned in this study are consistent with species-specific regulation of the LOX-HPL pathway during fruit ripening. For instance, raspberry samples were the only ripe fruit to contain *(Z)*-3-hexenal and had a much higher ratio of *(Z)*-3-isomers to *(E)*-2-isomers than the rest of the berry species analyzed. The expression of a hexenal isomerase gene has been strongly associated with the balance between *(Z)*-3 and *(E)*-2 isomers in woodland strawberry [35]. Therefore, the unique GLV isomer ratio in raspberry fruit samples may potentially reflect species-specific differences in the activities of downstream enzymes that utilize *(Z)*-3-hexenal as a substrate such as hexenal isomerase, which should be confirmed experimentally in future studies. Other GLVs demonstrated contrasting trends between species throughout the course of ripening, such as the decrease in *(Z)*-3-hexenyl acetate during ripening in blackberry samples, which generally increased during ripening in strawberry and raspberry samples. While AAT activity was not directly measured in this study, AATs are known to act as key enzymes in fruity ester biosynthesis during ripening, and variations in their gene expression and activity have been associated with changes in ester accumulation and aroma in several fruit species, including commercial strawberry, apricot, and grapes [36,37,38]. Alongside this result, the lower accumulation of hexyl acetate and *(E)*-2-hexenyl acetate in the ripe *Rubus* samples may indicate potential differences in the substrate specificity or activity of alcohol acyltransferases (AATs) or in the availability of their alcohol substrates. In contrast to canonical GLVs, the biosynthetic origins of *(E,E)*-2,4-hexadienal and 4-oxohex-2-enal remain poorly understood. Alongside *(Z)*-3-hexenal, these two putatively assigned compounds consistently decreased during ripening across all four species analyzed. In fruit, all three compounds formed a clear cluster in the hierarchical heatmap and shared the highest positive correlation of any GLVs analyzed. While these analyses were used as exploratory tools rather than statistically validated groupings, these three compounds display co-variation patterns, suggesting potential coordinated regulation during fruit development in berries from the *Rosaceae* family. Although *(Z)*-3-hexenal is a direct product of hydroperoxide lyases, *(E,E)*-2,4-hexadienal and 4-oxohex-2-enal may arise from downstream enzymatic or non-enzymatic transformations of canonical GLVs, which should also be validated in future experiments. Furthermore, as VOCs have been proposed as a tool for phenotyping processes such as ripening [39,40], the consistent decrease in these three compounds during ripening supports their further evaluation as candidate biomarkers for determining berry maturity. However, validation with non-invasive methods such as proton transfer reaction mass spectrometry (PTR-MS) and assessment of additional berry genotypes and growing locations are needed to validate the utility of GLVs as biomarkers.

Regarding organoleptic qualities, the differences in GLVs observed across ripening stages and species translated into predicted distinct aroma contributions. Due to the semi-quantitative nature of the analysis, OAVs were included to provide a qualitative indication of potential aroma relevance rather than precise quantitative measures of aroma impact. The predicted aroma contributions of GLVs in strawberry, raspberry, and blackberry samples were particularly distinct in the ripe stage, which is of greatest relevance to consumers. While OAVs only provide an estimate of aroma contribution, the higher number of odor-active GLVs and greater cumulative odor activity values seen in ripe strawberries suggest that GLVs contribute more strongly to the aroma of the strawberry samples when compared to the aroma of ripe raspberry or blackberry samples. Ester GLVs, especially hexyl acetate, had the strongest predicted influence on aroma in ripe strawberry samples, while, in contrast, aldehydes were predicted to be the most influential GLVs in ripe blackberry and raspberry samples. Esters are known to contribute to desirable fruity notes and are important determinants of strawberry aroma, while aldehydes are generally associated with less desirable herbaceous and grassy notes [41,42]. In all species analyzed, the predicted contribution of alcohol GLVs to aroma was shown to be minor in comparison with ester or aldehyde GLVs, as evidenced by their generally low odor activity values. Considering that *(E)*-2-hexenal and hexyl acetate have been associated with an increased perception of sweetness, the species-specific differences in these compounds may also contribute to differences in flavor perception [1].

Lastly, leaves of berry plants had considerably higher GLV abundances than fruit of the same species and lacked the esters *(E)*-2-hexenyl acetate and hexyl acetate. These patterns are consistent with tissue-specific regulation of the LOX–HPL pathway, though the underlying mechanisms were not directly investigated in this study. In previous studies, variations in phytohormonal regulation have been associated with tissue-specific volatile profiles and distinct ecological roles of GLVs in tritrophic interactions [43]. The detection of *(Z)*-3-hexenyl acetate in both fruit and leaf tissues is also consistent with findings in other Rosaceae species; this ester was the only one detected in fruit and leaves of quince, while other esters were only found in fruit [44]. When released from leaves, *(Z)*-3-hexenyl acetate has well-documented roles in plant defense and plant–plant communication in other species, including defense priming against biotic and abiotic stresses [6,21,23,45,46]. However, in fruit ecology, ester GLVs are broadly implicated as oviposition cues [47], while *(E)*-2-hexenal has been associated with defense responses in both fruit and leaf tissues of other species [48,49]. Whether these ecological functions of GLVs apply to the berry species examined here remains to be determined experimentally. Notably, blackberry leaves had a higher proportion of GLV alcohols compared with the other analyzed berry species. This could possibly be attributed to relatively higher alcohol dehydrogenase activity compared to the other species, though this was not directly assessed [50]. This pattern is also consistent with previous work demonstrating that healthy blackberry leaves emit more *(Z)*-3-hexen-1-ol than related Rubus species [33]. The alcohol-rich GLV profile of blackberry leaves may have ecological importance given the known roles of GLV alcohols as infochemicals in other plants, including triggering antennal responses in herbivores, attracting parasitic wasps, and plant–plant priming against pathogens [51,52,53], though, as with tissue-specific GLV differences, the ecological relevance of these species-specific profiles requires direct experimental investigation.

It must be noted that strawberry samples were grown at a different location than the raspberry and blackberry samples, harvested in different seasons due to their respective local horticultural management, and underwent distinct post-harvest handling procedures due to logistical challenges. Additionally, the invasive sample preparation performed before SPME analysis, such as tissue homogenization, salt addition, and heated incubation, may have distinctly influenced GLV accumulation in different tissues due to their wound responsiveness. These confounding variables related to fruit sampling represent a limitation for cross-species and cross-tissue comparisons and should be considered when interpreting the results. However, such treatments are commonly used prior to SPME analysis, and all samples were processed using the same methodology, allowing for relative comparisons between samples. Future experiments should consider utilizing non-invasive methods such as PTR-MS to further reduce artifacts related to sample preparation.

The exploratory comparisons performed in this study revealed key differences in GLV profiles in samples from several berry species, expanding knowledge of how these compounds evolve during ripening, how they may shape the aroma of berries, and how they differ between fruits and leaves. Additionally, this work informs future experiments seeking to elucidate the genetic control of GLVs in the *Rosaceae* family, identify useful biomarkers linked to berry fruit quality, and clarify the role of berry leaf GLVs in ecological interactions.

## 4. Materials and Methods

### 4.1. Plant Material and Sampling

Woodland strawberry (*Fragaria vesca cv*. “Reine de Vallées”) and commercial strawberry (*Fragaria* × *ananassa cv.* “Dover”, “Starlette”, & “Tudla”) were grown in the greenhouses of IRTA Torre Marimon, Caldes de Montbui, Spain (latitude 41°36′ N, longitude 2°10′ E, altitude 203 m above sea level). Plant clones were grown in 5 L pots containing a coco substrate and perlite mix. Strawberry fruits and leaves were sampled from a minimum of five plant clones between April and May 2024. At least five fruits were sampled per ripening stage for each plant clone, as well as at least five healthy leaves of varying sizes for each plant clone. Fruits of each species, line, and ripening stage were pooled to generate a representative composite pooled sample. The pooled sample representing each line was analyzed in three technical triplicates to assess reproducibility; thus, no independent biological replicates were analyzed; the same procedure was followed for leaves. The ripening stage of fruits was determined by color and days after pollination as follows: unripe corresponded with roughly 14–19 days after pollination and light green or white color, half-ripe corresponded with roughly 20–26 days after pollination and both white and pink color, and ripe corresponded with roughly 27–32 days after pollination and a full red color. Fruits and leaves were transported to the laboratory on ice, frozen and powdered in liquid nitrogen, and stored at −75 °C until analysis. Horticultural management, such as treatment with pesticides and herbicides, irrigation, and fertilizer application, was carried out as per local guidelines.

Blackberry (*Rubus* sp.) breeding lines RM-167, RM-215, and RM-276, as well as the raspberry (*Rubus idaeus*) breeding lines RF-055, RF-060, and RF-076, were grown in the greenhouses of PLANASA Cartaya, Huelva, Spain (latitude 37°18′ N, longitude 7°04′ W, altitude 51 m above sea level). Breeding line clones were products of distinct crosses to ensure genetic and phenotypic diversity and were grown as per local horticultural guidelines. Fruits and leaves were collected from a minimum of five plant clones between October and November 2024. Fruits and leaves were sampled, pooled, and analyzed as technical triplicates without biological replication, as described for the strawberry samples.

The ripening stage of fruits was determined by color as follows: unripe blackberries and raspberries corresponded with a majority green color, half-ripe blackberries corresponded with a majority red color, half-ripe raspberries corresponded with a majority pink color, ripe blackberries corresponded with a full purple color, and ripe raspberries corresponded with a full dark red color. Fruits and leaves were shipped overnight on ice the same day they were sampled, frozen and powdered in liquid nitrogen, and stored at −75 °C until analysis.

### 4.2. GC-MS Analysis of Green Leaf Volatiles

For analysis of green leaf volatiles in fruit and leaf samples, either 1 g of frozen powdered fruit or 100 mg of frozen powdered leaves was added to a 20 mL screw top GC-MS headspace vial, ensuring samples remained frozen at all times. Afterwards, 1 mL of a saturated NaCl solution containing 10 ppm 3-hexanone was added as an internal standard, and the vial was sealed using a metal screw top with a silicone/polytetrafluoroethylene (PTFE) septum.

Headspace vials were agitated at 1000 rpm using a Heatex Stirrer (CTC Analytics AG, Zwingen, Switzerland) and incubated at 50 °C for 10 minutes. Subsequently, a Smart SPME Arrow (Carbon-WR/PDMS, 1.1 mm diameter, 120 μm phase thickness; Restek, Bellefonte, PA, USA) penetrated the vial and extracted the volatiles in the headspace for 30 minutes at 50 °C and 1000 rpm. Extracted volatiles were desorbed at the GC injection port at 250 °C for 2 min, using a 5:1 split for fruit samples and a 20:1 split for leaf samples. Different sample masses and GC split ratios were used for fruits and leaves to maintain signal intensity within the linear dynamic range of the detector while avoiding SPME Arrow saturation, given the substantially higher GLV abundances in leaf tissue. Analytical comparability between tissue types is supported by normalization to the internal standard 3-hexanone, which undergoes identical extraction and injection conditions as the analytes and thus accounts for differences in sample mass and split ratio. Volatile compounds were analyzed by GC–MS using an Intuvo 9000 GC system paired to a 5977C MSD (Agilent Technologies, Santa Clara, CA, USA). A PAL System RSI 120 Sampler (CTC Analytics AG, Zwingen, Switzerland) was used for incubation, desorption, and extraction of volatiles. An HP-5MS UI GC Intuvo column (30 m, 0.25 mm, 0.25 μm; Agilent Technologies, Santa Clara, CA, USA) with a helium carrier gas flow of 1.2 mL/min was used for chromatographic separation of volatiles. The oven temperature started at 40 °C for 3 min, increasing by 2 °C/min ramp until reaching 50 °C, which was maintained for 1 min, then increased by 5 °C/min until 150 °C, which was maintained for 1 min, then increased by 40 °C/min until reaching 250 °C, which was maintained for 5 min. Regarding the mass spectrometry conditions, an electron ionization source was used with an ionization energy of 70 eV and a mass scan range from 30 u to 550 u.

Manual integration of peaks was performed using MassHunter Quantitative Analysis software (v.12.0) (Agilent Technologies, Santa Clara, CA, USA). To validate the identity of compounds, mass spectra and retention times were compared with those of commercial standards when available. Compounds were also validated by comparison with the NIST20 mass spectra library (Agilent Technologies, Santa Clara, CA, USA); compounds without authentic standards met a minimum library match factor threshold of 800. Furthermore, experimental linear Van den Dool-Kratz retention indices were determined with a series of *n*-alkanes (C7-C30, Sigma-Aldrich, St. Louis, MO, USA) run under identical chromatographic conditions, with a retention index tolerance window of ±10. Manually integrated peak areas were normalized by weight and comparison with the peak area of the internal standard, 3-hexanone. Normalized abundances are presented as μg kg^−1^ relative to 3-hexanone, a semi-quantitative approximation of concentration.

### 4.3. Determination of Odor Activity Values

Odor activity values (OAVs) were calculated by dividing the normalized abundance of a compound by its odor threshold. Given that OAVs were calculated using normalized abundances without compound-specific external calibration, OAVs should be interpreted as approximate and exploratory estimates rather than absolute measures of aroma influence. Nevertheless, as the same methodology was applied consistently across all samples, OAV comparisons between samples and species remain informative on a relative basis. Compounds with an OAV > 1 were considered to be potentially odor-active. Odor thresholds and aroma descriptors were sourced from the literature [6,10,54,55,56,57,58,59,60,61].

### 4.4. Chemicals and Reagents

Commercial standards of 3-hexanone (purity ≥ 98) were purchased from Thermo Fisher Scientific, Waltham, MA, USA. Commercial standards of *(E)*-2-hexenal (purity ≥ 98), *(Z)*-3-hexenal (50% in triacetin, stabilized), hexanal (purity ≥ 99%), *(E)*-2-hexen-1-ol (purity ≥ 96%), *(Z)*-3-hexen-1-ol (purity ≥ 99%), 1-hexanol (purity ≥ 99%), *(E)*-2-hexenyl acetate (purity ≥ 98%), *(Z)*-3-hexenyl acetate (purity ≥ 99%), and hexyl acetate (purity ≥ 99%) were purchased from Sigma-Aldrich (St. Louis, MO, USA).

### 4.5. Data Analysis

Data analysis was conducted in R (v4.5.1). For each sample, GLV abundances are reported as the mean ± standard deviation of three technical replicates of a single pooled composite sample to assess method precision. Each sample type was represented by a single pooled sample with no independent biological replicates; therefore, inferential statistical comparisons between groups (e.g., ripening stages, species, or tissues) were not performed. Multivariate analyses, including hierarchical clustering and principal component analysis (PCA), were performed in MetaboAnalyst 6.0. Spearman rank correlations between compounds were calculated to explore co-variation patterns. Heatmaps, bar charts, proportional stacked bar charts, and radar charts were generated in R (v4.5.1) as exploratory visualization tools.

## Figures and Tables

**Table 1:** Green leaf volatiles GC-MS Analysis. List of the VOCs detected with GC-MS that are classified as green leaf volatiles. Their linear retention indices, ions used for quantification, and compound family are listed below.

Compound Name	Retention Index (NIST)	Retention Index Deviation (Experimental vs. NIST)	Quantifier Ion (*m*/*z*)	Qualifier Ions (*m*/*z*)	Compound Family
*(Z)*-3-hexenal ^a^	801	801 (±0)	98	55, 69, 83	Aldehyde
Hexanal ^a^	802	803 (+1)	44	56, 72, 82	Aldehyde
*(Z)*-3-hexen-1-ol ^a^	851	851 (±0)	82	41, 55, 67	Alcohol
*(E)*-2-hexenal ^a^	854	847 (−7)	83	39, 69, 98	Aldehyde
*(E)*-2-hexen-1-ol ^a^	862	863 (+1)	57	39, 41, 67	Alcohol
1-hexanol ^a^	867	866 (−1)	56	43, 55, 84	Alcohol
*(E,E)*-2,4-hexadienal ^b^	915	910 (−5)	81	53, 67, 96	Aldehyde
4-oxohex-2-enal ^b^	958	965 (+7)	83	55, 57, 112	Aldehyde
*(Z)*-3-hexenyl acetate ^a^	1016	1015 (−1)	67	41, 43, 82	Ester
Hexyl acetate ^a^	1017	1022 (+5)	84	56, 61, 69	Ester
*(E)*-2-hexenyl acetate ^a^	1019	1025 (+6)	67	43, 82, 100	Ester

^a^ Commercial standard of compound, library match quality, and linear retention indices were used to confirm peak identity. ^b^ Library match quality and linear retention indices were used to putatively assign peak identity.

**Table 2:** List of the odor activity values calculated for ripening berries. OAVs calculated for unripe, half-ripe, and ripe developmental stages of fruit from various lines of strawberry (*F. vesca* & *F.* × *ananassa*), blackberry (*Rubus* sp.) and raspberry (*R. idaeus*). Values represent the mean ± standard deviation of n = 3 technical replicates of a single pooled sample. * Odor threshold units (µg kg^−1^).

Compound	*(Z)*-3-Hexenal	*(E)*-2-Hexenal	Hexanal	*(E,E)*-2,4-Hexadienal	*(Z)*-3-Hexen-1-ol	*(E)*-2-Hexen-1-ol	1-Hexanol	*(Z)*-3-Hexenyl Acetate	*(E)*-2-Hexenyl Acetate	Hexyl Acetate
Odor Threshold *	1.7	17	5	60	70	100	500	16	210	2
**Aroma Description**	Fatty, grassy	Green, grassy, fresh	Green, grassy	Sweet, green	Grassy, fresh	Green, fresh	Green, fresh	Fruity, herbal, banana	Fruity, apple, fresh	Fruity, apple, sweet
**Line**	***F. vesca cv. “Reine de Vallées”***									
**Unripe**	1318.07 ± 505.74	1405.42 ± 29.80	2480.55 ± 294.33	14.39 ± 4.89	13.17 ± 0.58	-	0.93 ± 0.05	3.97 ± 0.38	-	-
**Half-ripe**	603.20 ± 221.03	862.17 ± 135.51	752.60 ± 95.02	6.48 ± 2.19	7.93 ± 0.38	1.23 ± 0.21	0.88 ± 0.10	111.01 ± 23.31	5.72 ± 1.40	1066.84 ± 258.64
**Ripe**	-	527.35 ± 25.68	178.10 ± 24.85	-	2.00 ± 0.18	2.08 ± 0.28	0.68 ± 0.08	382.76 ± 15.91	59.14 ± 2.29	8800.62 ± 241.31
**Line**	***F.*** ** × ** ***ananassa cv. “Dover”***									
**Unripe**	188.59 ± 73.77	557.75 ± 103.04	121.86 ± 9.71	2.61 ± 1.03	2.55 ± 0.07	1.06 ± 0.06	0.88 ± 0.03	11.46 ± 0.25	1.66 ± 0.03	169.80 ± 5.07
**Half-ripe**	165.96 ± 55.26	699.22 ± 152.43	205.28 ± 19.97	1.92 ± 0.49	-	0.62 ± 0.08	0.11 ± 0.01	12.98 ± 1.21	1.44 ± 0.14	160.72 ± 20.81
**Ripe**	-	609.92 ± 36.01	118.45 ± 11.14	1.20 ± 0.12	-	1.07 ± 0.14	0.21 ± 0.03	77.82 ± 6.20	65.98 ± 4.28	2273.41 ± 165.52
**Line**	***F.*** ** × ** ***ananassa cv. “Starlette”***									
**Unripe**	868.66 ± 336.62	811.70 ± 280.09	245.80 ± 14.09	7.23 ± 1.94	5.70 ± 0.26	1.02 ± 0.11	1.30 ± 0.02	13.17 ± 0.84	2.68 ± 0.14	350.58 ± 16.18
**Half-ripe**	94.60 ± 50.76	162.73 ± 31.73	82.17 ± 4.30	0.89 ± 0.38	-	0.37 ± 0.02	0.06 ± 0.00	7.20 ± 0.09	1.11 ± 0.07	71.23 ± 1.68
**Ripe**	-	507.63 ± 101.68	109.11 ± 14.23	1.12 ± 0.08	-	2.02 ± 0.42	0.32 ± 0.05	136.12 ± 5.54	150.20 ± 1.70	5245.43 ± 60.41
**Line**	***F.*** ** × ** ***ananassa cv. “Tudla”***									
**Unripe**	459.82 ± 241.27	602.37 ± 178.43	219.55 ± 21.82	4.09 ± 2.23	2.67 ± 0.19	2.94 ± 0.19	0.88 ± 0.06	9.44 ± 0.58	4.59 ± 0.36	243.36 ± 32.91
**Half-ripe**	342.07 ± 172.57	1227.88 ± 172.47	278.74 ± 16.05	3.25 ± 1.32	1.86 ± 0.19	3.24 ± 0.41	0.68 ± 0.06	58.71 ± 2.93	34.79 ± 0.77	1510.94 ± 35.29
**Ripe**	-	472.06 ± 11.28	161.60 ± 16.05	1.09 ± 0.26	-	2.11 ± 0.17	0.29 ± 0.02	52.34 ± 1.54	32.43 ± 0.78	1088.99 ± 34.80
**Line**	***R. idaeus RF-055***									
**Unripe**	875.71 ± 113.07	261.94 ± 70.72	142.10 ± 9.62	6.04 ± 0.60	0.89 ± 0.18	-	0.02 ± 0.01	4.57 ± 0.14	-	-
**Half-ripe**	607.30 ± 77.31	148.30 ± 14.37	97.79 ± 5.07	4.44 ± 0.93	0.79 ± 0.07	-	0.02 ± 0.00	11.73 ± 0.78	-	-
**Ripe**	238.74 ± 14.74	48.86 ± 1.64	84.47 ± 3.54	1.23 ± 0.08	0.67 ± 0.07	-	0.03 ± 0.01	169.56 ± 2.82	0.20 ± 0.01	64.99 ± 2.13
**Line**	***R. idaeus RF-060***									
**Unripe**	1508.45 ± 312.04	402.58 ± 78.50	217.77 ± 14.99	9.87 ± 1.89	2.23 ± 0.16	-	0.02 ± 0.00	8.67 ± 0.29	-	-
**Half-ripe**	585.84 ± 65.11	124.12 ± 20.15	103.07 ± 11.57	3.34 ± 0.20	1.26 ± 0.34	-	0.03 ± 0.01	14.07 ± 2.44	-	-
**Ripe**	420.36 ± 22.15	79.40 ± 16.12	93.92 ± 4.77	1.93 ± 0.09	-	-	0.01 ± 0.00	170.81 ± 3.52	0.06 ± 0.01	67.43 ± 3.77
**Line**	***R. idaeus RF-076***									
**Unripe**	848.65 ± 78.85	191.90 ± 23.60	162.48 ± 2.68	6.78 ± 0.66	0.36 ± 0.10	-	0.01 ± 0.00	8.17 ± 0.18	-	-
**Half-ripe**	566.13 ± 32.37	106.01 ± 29.61	146.93 ± 7.70	3.43 ± 0.29	-	-	0.01 ± 0.00	13.50 ± 1.76	-	-
**Ripe**	121.33 ± 15.90	34.40 ± 5.94	75.04 ± 6.92	0.57 ± 0.06	-	-	0.01 ± 0.00	118.25 ± 4.58	-	84.71 ± 3.47
**Line**	***Rubus*** ** sp. ** ***RM-167***									
**Unripe**	449.80 ± 117.65	83.93 ± 4.50	205.61 ± 33.90	4.36 ± 1.82	1.85 ± 0.10	-	0.05 ± 0.01	1.50 ± 0.08	-	-
**Half-ripe**	151.62 ± 41.24	49.11 ± 17.00	122.72 ± 20.55	1.04 ± 0.27	-	0.08 ± 0.03	0.03 ± 0.01	0.49 ± 0.07	0.07 ± 0.01	-
**Ripe**	-	46.72 ± 4.49	48.42 ± 5.59	-	-	0.46 ± 0.08	0.11 ± 0.03	0.93 ± 0.15	0.82 ± 0.03	10.37 ± 0.51
**Line**	***Rubus*** ** sp. ** ***RM-215***									
**Unripe**	498.50 ± 118.78	112.64 ± 12.25	172.18 ± 20.87	4.88 ± 1.68	1.80 ± 0.15	-	0.04 ± 0.01	11.18 ± 0.78	-	-
**Half-ripe**	126.87 ± 28.45	53.99 ± 4.14	115.58 ± 13.10	0.91 ± 0.32	0.72 ± 0.06	0.11 ± 0.04	0.05 ± 0.02	3.45 ± 0.15	0.17 ± 0.01	-
**Ripe**	-	378.82 ± 106.57	223.14 ± 59.80	0.79 ± 0.20	-	1.52 ± 0.14	0.27 ± 0.03	0.93 ± 0.07	0.78 ± 0.09	12.53 ± 1.35
**Line**	***Rubus*** ** sp. ** ***RM-276***									
**Unripe**	229.01 ± 44.29	89.04 ± 35.12	252.63 ± 18.68	2.02 ± 0.50	0.59 ± 0.17	-	0.05 ± 0.01	3.61 ± 0.45	-	-
**Half-ripe**	299.06 ± 43.81	167.52 ± 47.98	291.54 ± 15.86	2.30 ± 0.39	0.74 ± 0.17	0.10 ± 0.06	0.05 ± 0.02	0.76 ± 0.10	0.04 ± 0.00	-
**Ripe**	-	255.90 ± 25.00	116.92 ± 27.60	0.39 ± 0.08	-	1.70 ± 0.28	0.39 ± 0.06	1.23 ± 0.13	0.95 ± 0.03	41.22 ± 2.82

## Data Availability

All data is available in the tables and figures within the manuscript and Supplementary Materials.

## Supplementary Materials

The following supporting information can be downloaded at: https://www.mdpi.com/article/10.3390/plants15111639/s1, Supplementary Table S1: Berry fruits and leaves GLV raw data. Supplementary Table S2: GLV totals, means. and SD. Supplementary Table S3: GLV proportional composition data. Supplementary Table S4: Compound correlation statistics. Supplementary Figure S1: PCAs of berry samples from all species at the same ripening stage.
