# Supplementary figures and images for: Green Leaf Volatile Profiling Reveals Ripening-Stage- and Tissue-Specific Patterns in Rosaceae Berries
Source: Plants (Basel). 2026 May 27;15(11):1639. doi: 10.3390/plants15111639 (PMC13258824; doi:10.3390/plants15111639)

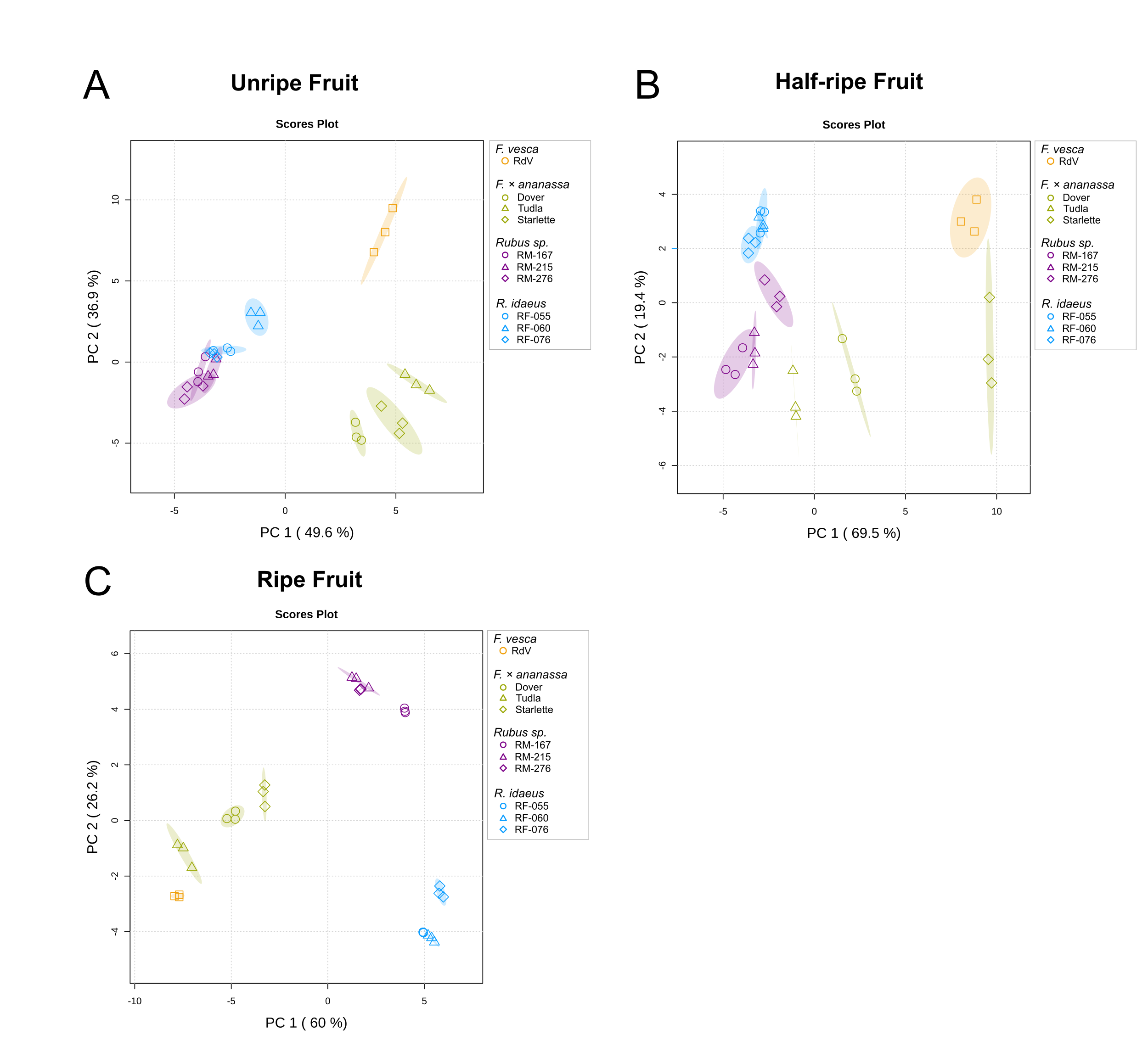

Supplement: Supplementary file 1 [file plants-15-01639-s001.zip › Fig S1 - PCA by ripening stages_600dpi revised.png]
